# Experiences and Needs Regarding Information Provision in Children With Haemophilia: A Qualitative Study on Caregivers’ and Healthcare Providers’ Perspectives

**DOI:** 10.1111/hae.70063

**Published:** 2025-05-19

**Authors:** Caroline M. A. Mussert, Nadia C. W. Kamminga, Evelien S. van Hoorn, Tjaisha M. Eekelaar, Silje R. Dehli, Carolien van der Velden‐van ‘t Hoff, Sasja Andeweg, Simone H. Reitsma, Annebelle C. M. Nooteboom, Adinda Diekstra, Armaĝan Albayrak, Marjon H. Cnossen, the SYMPHONY consortium

**Affiliations:** ^1^ Department of Pediatric Hematology and Oncology Erasmus MC Sophia Children's Hospital University Medical Center Rotterdam Rotterdam the Netherlands; ^2^ Department of Quality and Patient Care Erasmus MC University Medical Center Rotterdam Rotterdam the Netherlands; ^3^ Department of Dermatology Erasmus MC Cancer Institute University Medical Center Rotterdam Rotterdam the Netherlands; ^4^ Department of Public Health Erasmus MC University Medical Center Rotterdam Rotterdam the Netherlands; ^5^ Department of Human‐Centered Design Faculty of Industrial Design Engineering Delft University of Technology Delft the Netherlands; ^6^ Department of Design Organization and Strategy faculty of Industrial Design Engineering Delft University of Technology Delft the Netherlands; ^7^ NVHP for Everyone with a Congenital Bleeding Disorder (Dutch Patient Society) Nijkerk the Netherlands

**Keywords:** haemophilia, information provision, patient education, qualitative research, unmet needs

## Abstract

**Introduction:**

In haemophilia, ever more effective treatment options leading to minimal bleeding make information provision about the disease and its symptoms and when to alert the treatment team increasingly important. However, little is known about how current information provision is perceived and what the needs are.

**Aim:**

Gain in‐depth insights into experiences and needs regarding information provision of caregivers of young children with haemophilia, and the perspectives of their healthcare providers (HCPs).

**Methods:**

A qualitative study was conducted including 15 semi‐structured interviews with caregivers and seven interviews with HCPs. Purposive sampling ensured a varied sample regarding the child's age, type and severity of haemophilia, and treatment strategy. A comprehensive thematic content analysis was subsequently conducted using several phases of coding.

**Results:**

Three main themes were identified. First, caregivers and HCPs indicated that current disease knowledge and information provision regarding haemophilia varies and could be improved. Both groups underlined the importance of adequate information provision to support decision‐making and alleviate anxiety. Second, the need for standardized, centralized and tailored information was expressed, preferably digital. Current information is experienced as fragmented and incomplete, leading to lack of structure and uncertainties. Lastly, information provision cannot exist without additional coaching by the multidisciplinary treatment team and peers.

**Conclusion:**

Both caregivers and HCPs experience unmet needs regarding information provision as currently performed. Empowerment can be provided by standardized, centralized information tailored to disease severity and phase of life. A digital information platform with visual support, could provide a complete, up‐to‐date, readily available and reliable resource.

## 1 Introduction

The therapeutic landscape for patients with haemophilia has advanced immensely over the past years. Non‐factor replacement therapies have greatly improved quality of life [1] but have also reduced contact and communication with the haemophilia treatment center (HTC) due to less complex therapeutic regimes, less venous access problems and lower disease burden. Consequently, haemophilia plays a less prominent role in patients’ lives. Paradoxically, better disease knowledge, communication and self‐management skills are required and expected from these less experienced patients to avoid worsening of clinical outcomes.

Self‐management involves individuals actively managing their chronic illness by addressing physical, psychological and social aspects [2, 3]. In chronic conditions like haemophilia, self‐management is crucial to improve health behaviour and status and prevent hospitalization [4, 5]. Equally important is self‐efficacy—trust in one's own capacities to solve health issues [6]. Both capacities contribute to better long‐term health outcomes, reduced healthcare costs, and increased health‐related quality of life [4, 7].

Both effective self‐management and self‐efficacy require adequate disease knowledge and communication skills, which rely on education, information transfer from healthcare providers (HCPs), and access to high quality information [2, 8]. Effectively processing and retrieving medical information is essential for patient comprehension, proactive patient engagement and shared decision‐making [9], especially in chronic and rare diseases like haemophilia. However, studies show patients retain only 20%–60% of provided information due to the delivery method (oral/written), use of complex medical terminology, and stress [10, 11]. Especially patients with rare diseases face challenges when seeking information, as expertise and high‐quality information are more scarce [12].

According to the World Federation of Haemophilia guidelines, HCPs should provide comprehensive education to equip patients and caregivers with essential knowledge and skills [13]. Yet, a recent study investigating patients’ and HCPs’ perspectives on the quality of haemophilia care identified information provision as an area for improvement [14]. It is, however, unknown how current information provision is experienced and what specific needs are. Moreover, although disease education is stated as important [15], what the multidisciplinary healthcare team considers adequate information provision is not defined. Therefore, the aim of this qualitative interview study was to gain in‐depth insights into the experiences and needs regarding information provision of caregivers of young children with haemophilia, as well as the perspectives of their HCPs.

## 2 Materials and Methods

### 2.1 Study Design

A qualitative interview study design was chosen to gain an in‐depth understanding of experiences, needs and perspectives [16]. The consolidated criteria for reporting qualitative research (COREQ) were used as a checklist for reporting in this study [17]. This study was reviewed by the Medical Ethical Research Committee of the Erasmus MC, Rotterdam, the Netherlands (MEC‐2023‐0225), and determined not subject to the Medical Research Involving Human Subjects Act (WMO). All participants signed informed consent for study participation and to use their data for research purposes.

### 2.2 Participant Selection and Recruitment

Eligible participants were Dutch‐speaking caregivers of young children with haemophilia (0–10 years) and HCPs involved in haemophilia care. All were treated or worked in the HTC of the Erasmus MC Sophia Children's Hospital, Rotterdam, the Netherlands. This is one of the largest national expert centers for rare bleeding disorders, nationally certified and EAHAD accredited as a gold pin institution, and European Reference Network (ERN), for example, EuroBloodNet.

Purposive sampling ensured a varied sample of caregivers while considering the child's age, type of haemophilia (A or B), disease severity and treatment strategy. Potential participants were invited to participate by their treating physician. After expressing interest, caregivers were contacted by the researcher for study details. All members of the multidisciplinary paediatric treatment team were invited as HCPs. No incentives for participation were offered. Initially, seven caregivers signed up. After eight additional caregiver interviews and seven HCP interviews, saturation was reached—meaning that no new themes were identified [18]—leading to cessation of sampling.

### 2.3 Data Collection

Before semi‐structured interviews, caregivers received a sensitizing workbook to prepare for the interview, consisting of several questions regarding caregivers’ experiences and needs with (current) information provision to activate a stream of consciousness regarding these insights. Workbooks were sent to caregivers’ home addresses a few weeks before the interview and returned to researchers after completion. Completion was, however, optional. Interviews were conducted face‐to‐face in the hospital or online using Microsoft Teams, depending on participant preference, between 1, May 2023, and 30, March 2024, by one of the researchers (C.M.A.M., female medical doctor, or T.M.E., female industrial design student). Both were not directly involved in haemophilia care. In case both caregivers were interested in participating, a joint interview was performed. Interviews lasted around 60 min and were audio‐recorded. Caregiver‐ and HCP‐specific interview guides were used to structure discussions, which were developed based on literature [14, 15, 19, 20] and research team experiences. Topics included experiences with current information provision, information processing and passing, and preferences regarding optimal information provision (Supporting Information S1). After analysis of the first seven interviews, the caregiver interview guide was refined to emphasize preferences, future needs, and ideal manner and content of information provision.

### 2.4 Data Analysis

**FIGURE 1:**
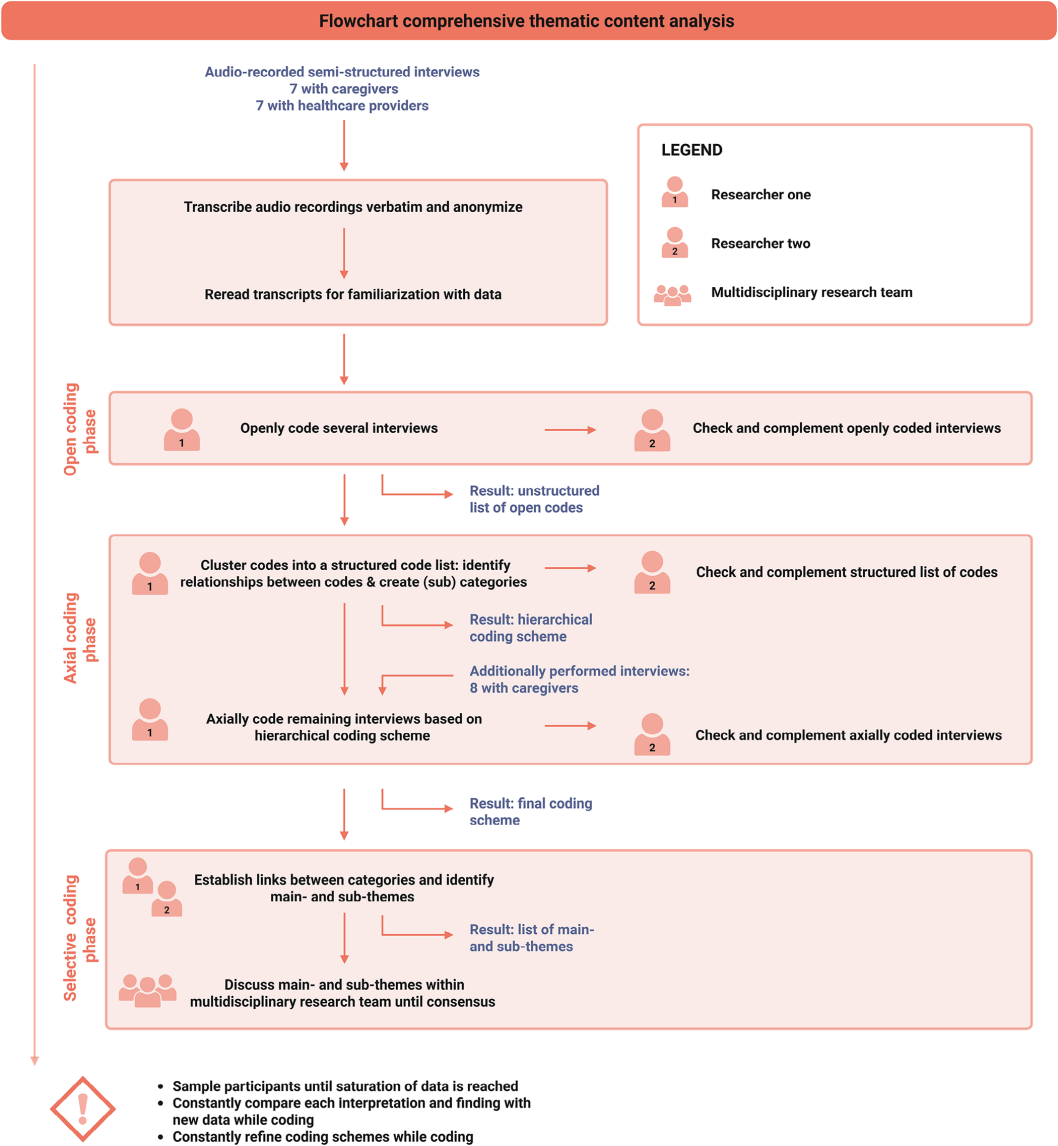
Flowchart of performed comprehensive thematic content analysis.

Audio recordings were transcribed verbatim and anonymized. Using NVivo version R1.7 (QSR International), a comprehensive thematic content analysis was performed [21, 22], using elements from grounded theory such as sampling until saturation, several phases of coding and constant comparison (Figure 1) [16]. First, transcripts were reread for familiarization. Subsequently, seven caregiver and three HCP interviews were openly coded by one researcher (C.M.A.M.) and checked and complemented by a second researcher (N.C.W.K., female medical doctor or E.S.v.H., female researcher), resulting in two unstructured lists of open codes. In the second, axial coding phase, codes were clustered into structured lists of codes by one researcher (C.M.A.M.) and reviewed and discussed with a second researcher (N.C.W.K.); relationships between codes were identified, and (sub)categories were created, resulting in two hierarchical coding schemes. The last eight caregiver and four HCP interviews were axially coded using these coding schemes by one researcher (C.M.A.M.) and checked and complemented by a second researcher (N.C.W.K.). Simultaneously, coding schemes were constantly refined. In the last, selective coding phase, interview data from both groups were compared to explore similarities and differences. Predominantly overlapping main and subthemes were identified, which were discussed within the multidisciplinary research team (C.M.A.M., N.C.W.K., M.H.C., female professor in paediatric haematology) until consensus was reached. Throughout the coding process, constant comparison was applied by comparing each interpretation and finding with new data [23].

## 3 Results

Table 1 presents caregivers characteristics and corresponding characteristics of their child. A total of 18 caregivers participated in 15 interviews. Table 2 shows characteristics of the seven participating HCPs.

**TABLE 1:** Characteristics of caregivers and their child with haemophilia undergoing study interviewing.

Number	Interview participants	Age^*^ (years)	Sex^*^	Type of haemophilia^*^	Disease severity^*^	Treatment strategy^*^
1	Mother, child	8	M	Haemophilia A	Severe	Prophylaxis with subcutaneous bispecific antibody
2	Mother	4	M	Haemophilia A	Severe	Prophylaxis with subcutaneous bispecific antibody
3	Mother, father, child	10	M	Haemophilia A	Mild	On‐demand
4	Mother, father	5	M	Haemophilia A	Mild	On‐demand
5	Mother	2	M	Haemophilia B	Severe	Prophylaxis with factor concentrate
6	Mother	8	M	Haemophilia A	Moderate	On‐demand
7	Mother	4 6	M M	Haemophilia A Haemophilia A	Mild Mild	On‐demand On‐demand
8	Mother	1	M	Haemophilia B	Severe	Prophylaxis with factor concentrate
9	Mother	6	M	Haemophilia B	Severe	Prophylaxis with factor concentrate
10	Mother	10	M	Haemophilia A	Mild	On‐demand
11	Mother, father	2	M	Haemophilia A	Severe	Prophylaxis with subcutaneous bispecific antibody
12	Mother	4	F	Haemophilia A	Mild	On‐demand
13	Mother, child	9	M	Haemophilia A	Moderate	Prophylaxis with subcutaneous bispecific antibody
14	Mother	4	M	Haemophilia A	Mild	On‐demand
15	Mother	9	F	Haemophilia A	Mild	On‐demand

*Note*: Children with severe haemophilia and one child with moderate haemophilia received prophylaxis with a subcutaneous bispecific antibody or factor concentrates to prevent bleeding. Children with moderate and mild haemophilia received on demand therapy with intravenous factor concentrates when bleeding occurred or to prevent bleeding in cases of head trauma or medical procedures. Two girls were (obligate) haemophilia carriers and patients (factor levels < 0.50 IU/mL).

* Age, sex, type of haemophilia, disease severity and treatment strategy refers to the child.

Abbreviations: F: female; M: male.

**TABLE 2:** Characteristics of healthcare providers undergoing study interviewing.

Number	Role in team	Gender	Years of experience	Intensity of involvement in haemophilia care
1	Nurse consultant	Female	> 5	Daily
2	Paediatric hematologist	Female	> 5	Daily
3	Paediatric physiotherapist	Female	> 3	Weekly
4	Nurse practitioner	Female	> 5	Daily
5	Fellow paediatric hematology	Female	> 1	Daily
6	Paediatric hematologist	Female	> 5	Daily
7	Social worker	Female	> 3	Weekly

**FIGURE 2:**
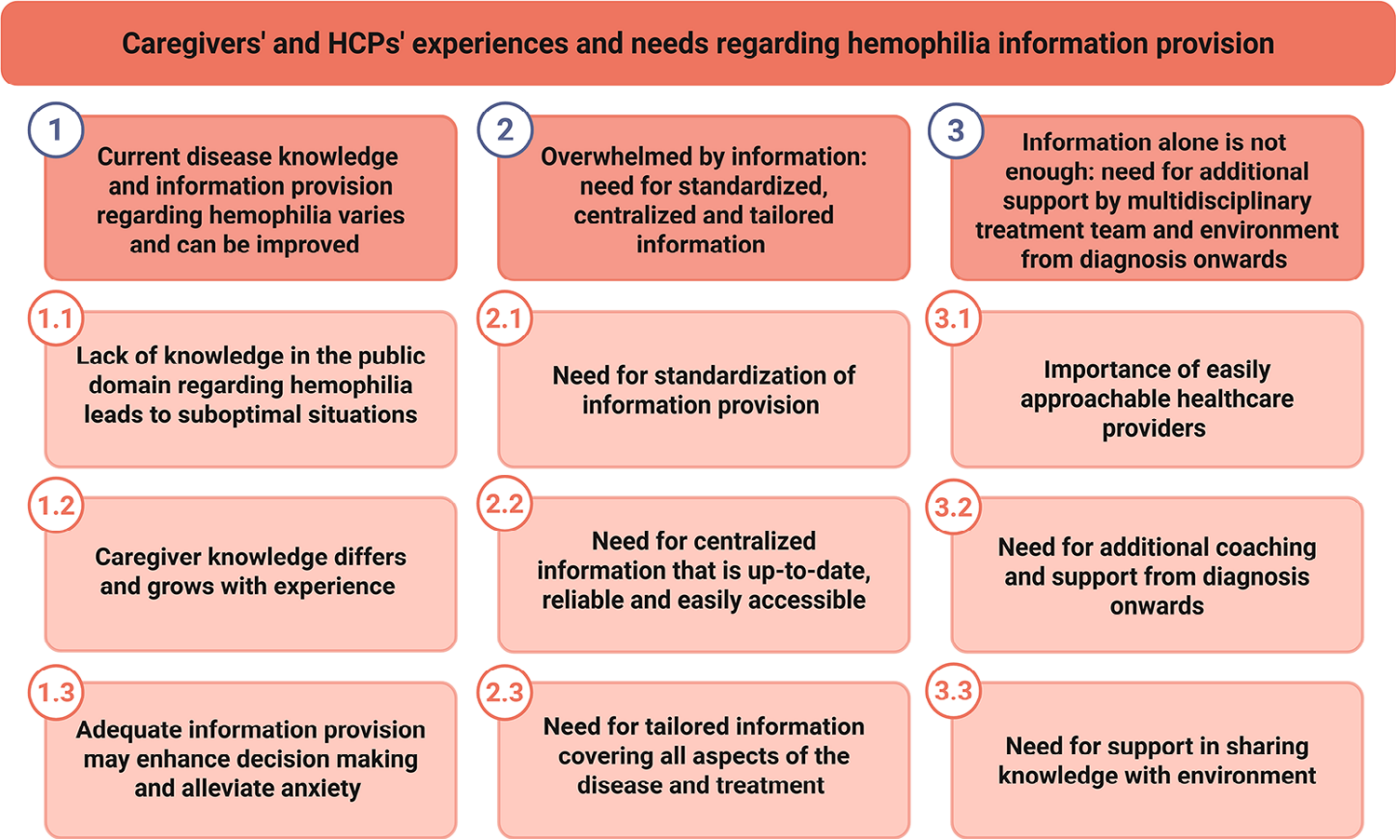
Overview of main and subthemes derived from study interviews.

**FIGURE 3:**
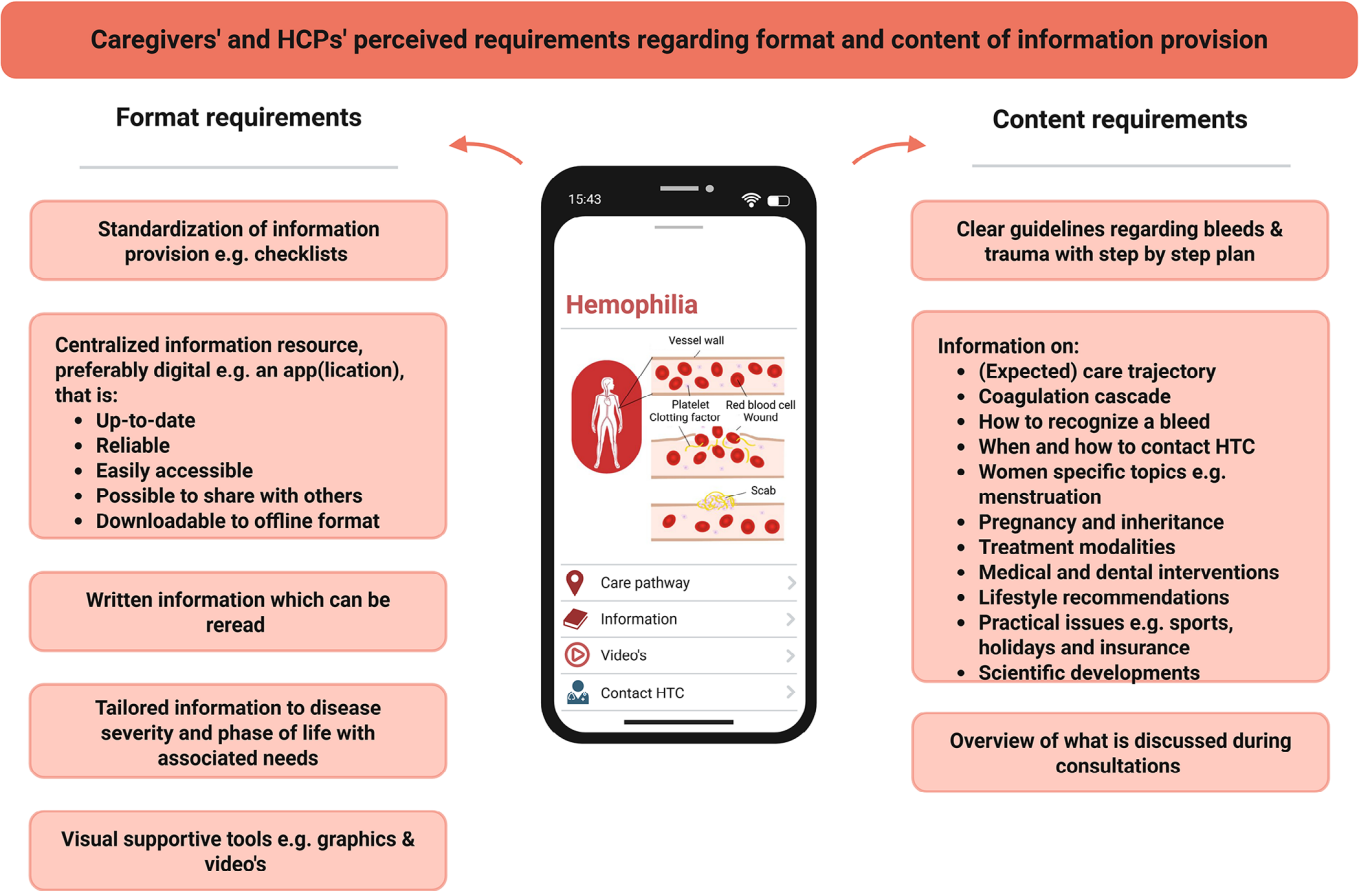
Overview of identified needs and preferences regarding future information provision.

Analysis resulted in three main themes and nine subthemes (Figure 2). Unless mentioned otherwise, subthemes apply to both caregivers and HCPs. Figure 3 provides an overview of identified perceived requirements regarding information provision.

### 3.1 Current Disease Knowledge and Information Provision Regarding Haemophilia Varies and Can Be Improved

The first main theme identified focused on disease knowledge and information provision regarding haemophilia, which was reported to vary and to be improved. This main theme was subdivided into three subthemes. Illustrative quotes are presented in Table 3.

**TABLE 3:** Illustrative quotes to main theme 1: Current disease knowledge and information provision regarding haemophilia varies and can be improved.

Subthemes	Quotes
1.1 Lack of knowledge in the public domain regarding haemophilia leads to suboptimal situations	*At the daycare center, they began to worry, another bruise…. They immediately thought: child abuse–* Haemophilia A, severe (CG11)
	*He [the medical specialist in regional hospital] told us our son had haemophilia A, the severe form. At the time, he didn't know exactly how severe it was, but he told me our son would not live past 30 years, and that he would soon end up in a wheelchair–* Haemophilia A, severe (CG1)
1.2 Caregiver knowledge differs and grows with experience	*Directly after diagnosis, you don't really understand what is being said. You wonder what they mean when someone tells you ‘the scab forms but the wound keeps bleeding underneath’, and you can't really picture a spontaneous joint bleed either […] It's only after experiencing it for the first time, that you truly understand what is being said–* Haemophilia A, severe (CG1)
	*After a while, you might think, ‘Oh, they know it all.’ But if you check their disease knowledge explicitly, you realize: 'Oh, wait, there's still a piece of information missing’*–Fellow paediatric hematology (HCP5)
1.3 Adequate information provision may enhance decision making and alleviate anxiety	*We're not dealing with it every day anymore […] so far, nothing serious has happened, so we may be starting to become a bit too relaxed about it all*–Haemophilia A, mild (CG10)

#### 3.1.1 Lack of Knowledge in the Public Domain Regarding Haemophilia Leads to Suboptimal Situations

Caregivers experienced a general lack of knowledge regarding haemophilia in the public domain, often causing suboptimal situations. This unfamiliarity led to anxiety, for example, amongst daycare and school and close relatives, making them reluctant to take care of their child. Moreover, caregivers experienced a lack of knowledge among HCPs not working in an HTC. Some caregivers shared that—when pregnant—they were not referred to an HTC despite informing the midwife about the family history of haemophilia, resulting in home births without necessary precautions. Also, general practitioners or paediatricians in regional hospitals tended to be less aware of how to diagnose a bleeding disorder when confronted with bleeding symptoms, sometimes leading to a delayed or incorrect diagnosis and unnecessary risks. This lack of knowledge also resulted in the exchange of incorrect information. In addition, some caregivers were suspected of child abuse.

Caregivers emphasized the importance of attentiveness and assertiveness to secure optimal care for their child. They reported it was regularly necessary to explain haemophilia's consequences and the importance of acting and treating promptly when visiting other departments in the Academic hospital, sometimes feeling as if their expertise regarding their child's health was not appreciated or ignored. HCPs acknowledged this general lack of knowledge outside the HTC and underlined the importance of increasing awareness and improving disease education.

#### 3.1.2 Caregiver Knowledge Differs and Grows With Experience

Both HCPs and caregivers noted that knowledge among caregivers varies greatly and grows over time. Initially, provided information such as how to recognize a bleed or when to call the HTC after a trauma is difficult to internalize but becomes clearer through real‐life experiences.

Furthermore, caregivers reported that recognizing a bleed and responding to acute situations becomes easier, yet applying acquired knowledge in daily life remains challenging. Caregivers mentioned that having a medical background or family history of haemophilia is beneficial. However, some noticed they still tended to underestimate acute situations. Therefore, HCPs emphasized the importance of checking caregivers’ understanding throughout the lifelong healthcare cycle.

#### 3.1.3 Adequate Information Provision May Enhance Decision‐Making and Alleviate Anxiety

HCPs highlighted that adequate knowledge of haemophilia and its symptoms enhances medical decision‐making in acute situations such as bleeding or trauma. They also noted that advances in treatment, leading to minimal bleeding, may in the long run lead to suboptimal recognition of early signs of bleeding and inadequate responses in acute, sometimes life‐threatening situations. HCPs stressed that repeating disease information helps to keep haemophilia on top of mind, which is especially important for non‐severe haemophilia and milder bleeding phenotypes. Caregivers confirmed that their focus on haemophilia is diminishing, as it plays a less prominent role in daily life.

HCPs also indicated that adequate information provision can help caregivers to share relevant information with their environment. Both HCPs and caregivers noted that this could reduce uncertainty and stress for both caregivers and close relatives (see also Subtheme 1.1).

### 3.2 Overwhelmed by Information: Need for Standardized, Centralized and Tailored Information

The second identified main theme concerned the need for standardized, centralized and tailored information, which was subdivided into three subthemes. Illustrative quotes are presented in Table 4.

**TABLE 4:** Illustrative quotes to main theme 2: Overwhelmed by information: Need for standardized, centralized and tailored information.

Subtheme	Quotes
2.1 Need for standardization of information provision	*In my experience, it is [information provision] not consistent. Sometimes I give an extensive explanation, other times I just refer them to the Cyberpoli [informative website for adolescents and young adults] website and the NVHP [Dutch patient society] […] Although I aim to do it the same way every time […] I'd prefer having a consistent methodology to explain everything to our patients. In this way, I'd know if I am forgetting something. Having a format to make sure we cover everything would be handy*–Nurse practitioner (HCP1)
2.2 Need for centralized information that is up‐to‐date, reliable and easily accessible	*I think an app would be great, which you can share with friends and family […] In this way you have an integrated site where all the information is located […] I think it is smart to develop something that is easy to update, and contains information that remains relevant*–Haemophilia B, severe (CG9)
	*Through a drawing, she [hematologist] showed how a wound heals, making it easier and clearer to understand. It really sticks with you in that way–* Haemophilia A, mild (CG14)
2.3 Need for tailored information covering all aspects of the disease and treatment	*The information you find on Google or whatever, you really have to check if it's also for mild haemophilia. There's a lot more about severe haemophilia […] So yeah, you run into that problem sometimes. I can filter it myself, but I can imagine some parents struggle with that*–Haemophilia A, mild (CG10)

#### 3.2.1 Need for Standardization of Information Provision

Although caregivers were generally positive about the information provided by HCPs, both groups indicated that information provision can be improved. First, both caregivers and HCPs stressed the importance of standardization. Caregivers indicated that receiving contradictive information from different HCPs led to confusion and uncertainty, with some realizing they missed information on certain topics. HCPs acknowledged this, as most information is provided verbally and not always supported by educative materials. Although HCPs indicated that certain standard topics are discussed, a standardized methodology for consistent information would be valuable. They also recommended giving caregivers insight into discussed topics beforehand, enabling preparation of consultations and formulation of questions. Caregivers agreed this would be valuable. In addition, as caregivers find it difficult to respond adequately in acute situations (see also Subtheme 1.2), they emphasized the need for a clear step‐by‐step plan when responding to bleeding or trauma and when and how to contact the HTC.

#### 3.2.2 Need for Centralized Information That Is Up‐to‐Date, Reliable, and Easily accessible

Caregivers and HCPs emphasized the fragmentation of information, scattered over numerous online and offline resources. As a result, it is not always clear which resources are reliable, answers to specific questions are difficult to find and not quickly accessible, and information is often incomplete, according to caregivers. HCPs also indicated that leaflets become rapidly outdated due to expanding therapeutic options. Therefore, both groups highlighted the need for a more centralized information resource that is up‐to‐date, reliable and easily accessible, also for others. Additionally, caregivers indicated that having information which can be reread is especially beneficial, as most information is provided verbally and is hard to recall. Both caregivers and HCPs suggested a digitalized and centralized resource such as an app(lication), with the possibility to download information to an offline format and share information with relatives.

According to HCPs, the centralization should be organized nationally, possibly by the patient society. Moreover, to facilitate usability and inclusivity, a need for more visual supportive tools (e.g., graphics or videos) was reported. Caregivers appreciated HCPs drawing during consultations as this increased their understanding and recall.

#### 3.2.3 Need for Tailored Information Covering All Aspects of the Disease and Treatment

Caregivers underscored the need for tailored information. They noted that information needs vary between disease severities. Caregivers of children with mild haemophilia noticed that information is often focused on severe haemophilia and does not apply to their own situation. Moreover, caregivers expressed a desire for information tailored to their child's phase of life and associated concerns, with the option to read ahead and prepare for relevant future topics, like menstruation and sexuality. Caregivers also mentioned that information could be more child‐friendly.

However, information needs differ per individual. Whereas some caregivers desired detailed information, others preferred to take things as they come. Yet, all caregivers expressed a need for practical information and advice on daily issues like sports participation and holiday preparation.

### 3.3 Information Alone is Not Enough: Need for Additional Support by Multidisciplinary Treatment Team and Environment From Diagnosis Onwards

The third identified main theme concerned the need for additional support, of which illustrated quotes are presented in Table 5. This theme was subdivided into three subthemes, of which the third mainly applied to caregivers.

**TABLE 5:** Illustrative quotes to main theme 3: Information alone is not enough: Need for additional support by multidisciplinary treatment team and environment from diagnosis onwards.

Subtheme	Quotes
3.1 Importance of easily approachable healthcare providers	*That one time it was a Sunday, and we had just gotten to know about haemophilia. And then, well, you feel kind of hesitant because it's Sunday evening, you know, and you think, should I really call?*–Haemophilia B, severe (CG5)
3.2 Need for additional coaching and support from diagnosis onwards	*With all the emotions and stress, not everything stuck […] hearing the diagnosis is a huge shock for many people. So, I think a follow‐up session or two would be really helpful for parents like me to process everything and feel more confident about the information–* Haemophilia A, mild (CG14)
3.3 Need for support in sharing knowledge with environment	I: *How do you feel about having to pass on information to others?* CG: *Stressful, extremely stressful, I can tell you, especially after what we experienced at school [teachers and parents acting overprotectively]. I thought, how am I going to formulate this? Because, I don't want to scare people, but I do want them to understand that it's really important. So that's quite difficult for me*–Haemophilia A, mild (CG14)

#### 3.3.1 Importance of Easily Approachable HCPs

Caregivers communicated that they saw their HCPs as a very valuable and reliable resource of information. They indicated a preference for contacting their HCPs for trustworthy, tailored and rapid answers rather than searching online. Both caregivers and HCPs emphasized the importance of the HTC's easy approachability. However, while caregivers appreciated knowing they could always contact their HCPs, some still felt burdened to do so. Caregivers suggested maintaining easy contact during office hours and suggested having an easily accessible and specialized contact person outside office hours.

#### 3.3.2 Need for Additional Coaching and Support From Diagnosis Onwards

Caregivers emphasized their need for additional coaching and support from diagnosis onwards, including peer support and psychosocial support, a sentiment acknowledged by HCPs. While caregivers appreciated the warm‐hearted consultations with their HCPs, they often found the first consultation after diagnosis overwhelming due to the large amount of information and associated emotions, causing insufficient retention of the provided information. Therefore, caregivers and HCPs suggested an additional consultation (by telephone) to reinforce and verify comprehension would be valuable, which is already done in some cases.

Furthermore, caregivers expressed a need for peer support to share experiences and practical questions. Peer support during pregnancy could help them prepare and assess the impact haemophilia will have on their child. However, some caregivers stressed the importance of careful peer‐matching, as differing disease severities can affect the usefulness of the support.

HCPs also highlighted the need for more psychosocial support, advocating for a bigger role for social workers and psychologists. They emphasized the added value of fixed contact moments at developmental milestones to identify challenges caregivers and children experience and to decrease the taboo of supportive care.

#### 3.3.3 Need for Support in Sharing Knowledge With Environment

Caregivers expressed a need for support in sharing knowledge with their environment, as this was felt to be stressful. Effective communication with others was experienced to be difficult due to the initial mostly verbal communication, which they often did not retain (see also Subtheme 3.2). Accordingly, how this information is shared influences how risks are calculated by close relatives, schools or in sports settings, leading to either overprotectiveness or inadequate responses in acute situations. To specifically support sharing of information, caregivers suggested the development of a platform with easily understandable information, supported by visuals (see also Subtheme 2.2).

## 4 Discussion

To our knowledge, this is the first qualitative study on caregivers’ and HCPs’ experiences regarding haemophilia information provision in a large, (inter)nationally accredited HTC. We included a varied sample of caregivers and HCPs, ensuring maximum variation in experiences [24]. Furthermore, caregiver selection was based on different disease characteristics of their child, including caregivers of children (including girls) with moderate and mild haemophilia, who are often under‐represented in studies [25, 26]. Our rigorous qualitative methodology, including a comprehensive thematic content analysis with, for example, multiple phases of coding [16, 21, 22], ensures an in‐depth understanding of experiences and needs. Reaching saturation further strengthens the validity of our results [18].

Caregivers and HCPs highlighted that the immense amount of public information is challenging to manage and filter. Moreover, patients often only partially remember medical information provided by HCPs, with recall often influenced by emotions [10, 11]. This was particularly noted when new information was provided verbally. To improve understanding and recall of health information, centralizing information, for example, by dissemination through the national patient society, and providing written resources for reference are crucial [27, 28]. In line with current healthcare digitalization [29], developing an app(lication) that provides a complete, up‐to‐date and reliable source of information was proposed. This aligns with patients’ preferences for digital information [14, 20, 30].

Another method to improve understanding and recall abilities is visual support, such as videos with spoken text and infographics alongside textual information [10, 31‐33], which was proposed and expressed as helpful by caregivers and HCPs. Moreover, visualized information enhances inclusivity, benefiting patients with lower health literacy skills [10, 32, 34]. As limited health literacy negatively influences patient outcomes [35, 36], this approach could improve self‐management, enhance patient empowerment, and facilitate shared decision‐making [9, 37, 38], resulting in better long‐term health outcomes.

Another important finding is the perceived unfamiliarity of haemophilia among HCPs outside HTCs and the general population, which was also highlighted in previous research [14, 19]. Increased awareness is essential for early diagnosis and adequate disease management [13]. Medical education for general practitioners and paediatricians should emphasize recognizing bleeding disorder (alarm) symptoms.

A limitation of our study is sampling from a single HTC and the age range of the children whose caregivers were included. However, as haemophilia care is centralized in the Netherlands and follows national guidelines [39], we believe most findings apply to other Dutch HTCs and similar settings abroad. Since haemophilia is often diagnosed in early childhood, when extensive information is provided, our identified themes are likely generalizable to other groups of haemophilia patients. Our sample included solely Dutch‐speaking caregivers—mostly mothers—and health literacy was not quantified, which is important as digital health tools may cause disparities between persons with varying health literacy [40]. Moreover, although mothers are traditionally more involved in child (health)care [41], including the fathers’ perspective could provide additional valuable insights. Future research should therefore include participants with diverse literacy levels, age groups and other bleeding disorders, as well as more male participants.

## 5 Conclusion

Although caregivers generally appreciate the information they receive, both caregivers and HCPs emphasized the need to increase awareness of haemophilia and improve information provision. To address these needs, information should be standardized, centrally available, and tailored to disease severity and phase of life, covering the complete patient journey. A digital information platform, such as an app(lication), with visual support could serve as a complete, up‐to‐date, and reliable resource that is readily available and enables sharing of information. Combined with additional support from a dedicated multidisciplinary treatment team, such a tool can empower patients, maintaining or improving patient outcomes despite the decrease of contact and communication moments.

## Author Contributions

C.M.A.M., T.M.E., and M.H.C. designed the study and E.S.v.H., A.A., and S.R.D. critically revised the design of the study. M.H.C., S.A., and C.v.d.V‐v.H. helped with patient selection. C.M.A.M. and T.E. collected the data and C.M.A.M., N.C.W.K., and E.S.v.H. performed the data analysis. C.M.A.M., N.C.W.K., and M.H.C. interpreted the data. C.M.A.M. wrote the manuscript together with N.C.W.K.; all other authors critically revised the manuscript. This manuscript was approved for submission by all authors.

## Ethics Statement

This study was reviewed and determined not to fall under the Medical Research Involving Human Subjects Act (WMO) by the Medical Ethical Research Committee of the Erasmus Medical Center, Rotterdam, the Netherlands (MEC‐2023‐0225).

## Consent

All participants signed informed consent to participate in the study and to use their data for research purposes.

## Conflicts of Interest

M.H. Cnossen has received grants from governmental and societal research institutes, for example, NWO‐ZonMW, NWO‐NWA, Innovation fund, and unrestricted investigator initiated research grants from private funds, as well as institutional grants, trial funding/educational and travel funding from the following companies over the years: Pfizer, Baxter/Baxalta/Shire Takeda, Bayer Schering Pharma, CSL Behring, Sobi Biogen, Novo Nordisk, Novartis, Nordic Pharma, Roche and het Sikkelcelfonds, and has served as a member on steering boards of Roche and Bayer. All grants, awards and fees go to the Erasmus MC as an institution. All other authors have no competing interest relevant to the contents of this manuscript.

## Supporting information



Supporting Information

## Data Availability

The data that support the findings of this study are available on request from the corresponding author. The data are not publicly available due to privacy or ethical restrictions.
