# Supplementary material for: Experiences and Needs Regarding Information Provision in Children With Haemophilia: A Qualitative Study on Caregivers’ and Healthcare Providers’ Perspectives
Source: Haemophilia. 2025 May 19;31(4):657–67. doi: 10.1111/hae.70063 (PMC12306852; doi:10.1111/hae.70063)
Supplement: Supplementary file 1 — Supporting Information [file HAE-31-657-s001.docx]

**Supplement 1. Topic guide experiences and needs regarding hemophilia information provision**

Introduction

- Background and aim of interview study
- Informed consent and consent for audio-recording
- Introduction participant including general characteristics disease child

Experiences regarding current information provision

- Experiences with diagnosis and (gaining) disease knowledge
- Experiences with existing information recourses and pros/cons
- Experiences with regular consultations and contact with healthcare providers

Experiences with hemophilia in social environment

- Experiences regarding sharing information with others e.g. family, friends, daycare, school, sports teachers
- Experiences with going on holidays
- Experiences with peer contact

Experiences with disease education of child

- Disease knowledge of child
- Experiences or expectations regarding disease education process of child

Future perspectives on information provision

- Experienced pain points with current information provision
- Preferences and needs regarding hemophilia information provision
- Thoughts on ideal information provision and information resource
